# Molecular subtyping of glioblastoma based on immune-related genes for prognosis

**DOI:** 10.1038/s41598-020-72488-4

**Published:** 2020-09-23

**Authors:** Xueran Chen, Xiaoqing Fan, Chenggang Zhao, Zhiyang Zhao, Lizhu Hu, Delong Wang, Ruiting Wang, Zhiyou Fang

**Affiliations:** 1 Anhui Province Key Laboratory of Medical Physics and Technology, Institute of Health and Medical Technology, Hefei Institutes of Physical Science, Chinese Academy of Sciences, No. 350, Shushan Hu Road, Hefei, 230031 Anhui China; 2 Department of Molecular Pathology, Hefei Cancer Hospital, Chinese Academy of Sciences, No. 350, Shushan Hu Road, Hefei, 230031 Anhui China; 3 The First Affiliated Hospital of USTC, Division of Life Sciences and Medicine, University of Science and Technology of China (USTC), No. 17, Lujiang Road, Hefei, 230001 Anhui China; 4 Department of Anesthesiology, Anhui Provincial Hospital, No. 17, Lujiang Road, Hefei, 230001 Anhui China; 5 University of Science and Technology of China, No. 96, Jin Zhai Road, Hefei, 230026 Anhui China

**Keywords:** Cancer microenvironment, CNS cancer, Tumour biomarkers, Tumour immunology

## Abstract

Glioblastoma (GBM) is associated with an increasing mortality and morbidity and is considered as an aggressive brain tumor. Recently, extensive studies have been carried out to examine the molecular biology of GBM, and the progression of GBM has been suggested to be correlated with the tumor immunophenotype in a variety of studies. Samples in the current study were extracted from the ImmPort and TCGA databases to identify immune-related genes affecting GBM prognosis. A total of 92 immune-related genes displaying a significant correlation with prognosis were mined, and a shrinkage estimate was conducted on them. Among them, the 14 most representative genes showed a marked correlation with patient prognosis, and LASSO and stepwise regression analysis was carried out to further identify the genes for the construction of a predictive GBM prognosis model. Then, samples in training and test cohorts were incorporated into the model and divided to evaluate the efficiency, stability, and accuracy of the model to predict and classify the prognosis of patients and to identify the relevant immune features according to the median value of RiskScore (namely, Risk-H and Risk-L). In addition, the constructed model was able to instruct clinicians in diagnosis and prognosis prediction for various immunophenotypes.

## Introduction

Glioblastoma (GBM), an aggressive primary malignancy in the central nervous system, has a median survival time of 12–15 months and a 5-year survival rate of < 5%^1,2^. According to the Clinical Practice Guideline formulated by the American National Comprehensive Cancer Network, chemotherapy is still the preferred choice for stage III–IV GBM^3^. Currently, there are various chemotherapy regimens, but some patients do not benefit from chemotherapy, and imaging examination could be applied to examine cancer development. In addition, the distinct long-term clinical outcomes may be detected based on tumor heterogeneities among these cases with the same pathological subtype^4^. However, some problems remain to be solved: how to assess tumor heterogeneities prior to treatment for these cases in a non-invasive or less traumatic way, estimate the risk of cancer progression, evaluate tumor response to chemotherapy in individual patients, and to estimate the different long-time overall survival (OS) among groups with different cancer heterogeneities^5^.

Currently, an immune disorder that can promote tumor genesis has been recognized as the enabling feature in the glioma genesis process^6^. Glioma cells can remarkably induce an immune response; in some cases, they can subjugate such a response to establish an appropriate microenvironment to promote their development^7^. Standard treatment cannot achieve a satisfying effect; thus, immunotherapy is being intensively investigated as an additional method^8^. Meanwhile, some parameters related to immunity have been reported to predict the disease prognosis, which has highlighted the significance of different immune states in identifying glioma outcomes^9,10^. Nonetheless, immune phenotypes in a glioma microenvironment, together with their relationship with prognosis, are rarely examined systemically.

Biomarkers are able to accurately estimate disease prognosis and patient survival, which are thereby valuable for decision-making in clinical GBM treatment^11,12^. Recently, an increasing number of studies have suggested that the expression patterns of genes can predict and classify the survival outcomes of GBM patients^13^. Nonetheless, this proposal has still not been identified as a clinical routine practice, which may be related to the lack of evidence, small sample size, and tremendous data fitting in most studies. Consequently, use of large-scale databases that are accessible to the public and involve the expression patterns of genes, like TCGA, makes it possible to identify the most reliable biomarkers to predict and classify GBM prognosis. In this study, a model to predict the prognosis of GBM was constructed and verified based on immune-related genes, according to the clinical characteristics of patients extracted from the ImmPort and TCGA databases. Our results can help clinicians evaluate the efficacy, predict the disease prognosis, and select the suitable GBM treatment.

## Results

### Mining of specific immune-related genes based on GBM patient survival and prognostic outcomes

**Figure 1:**
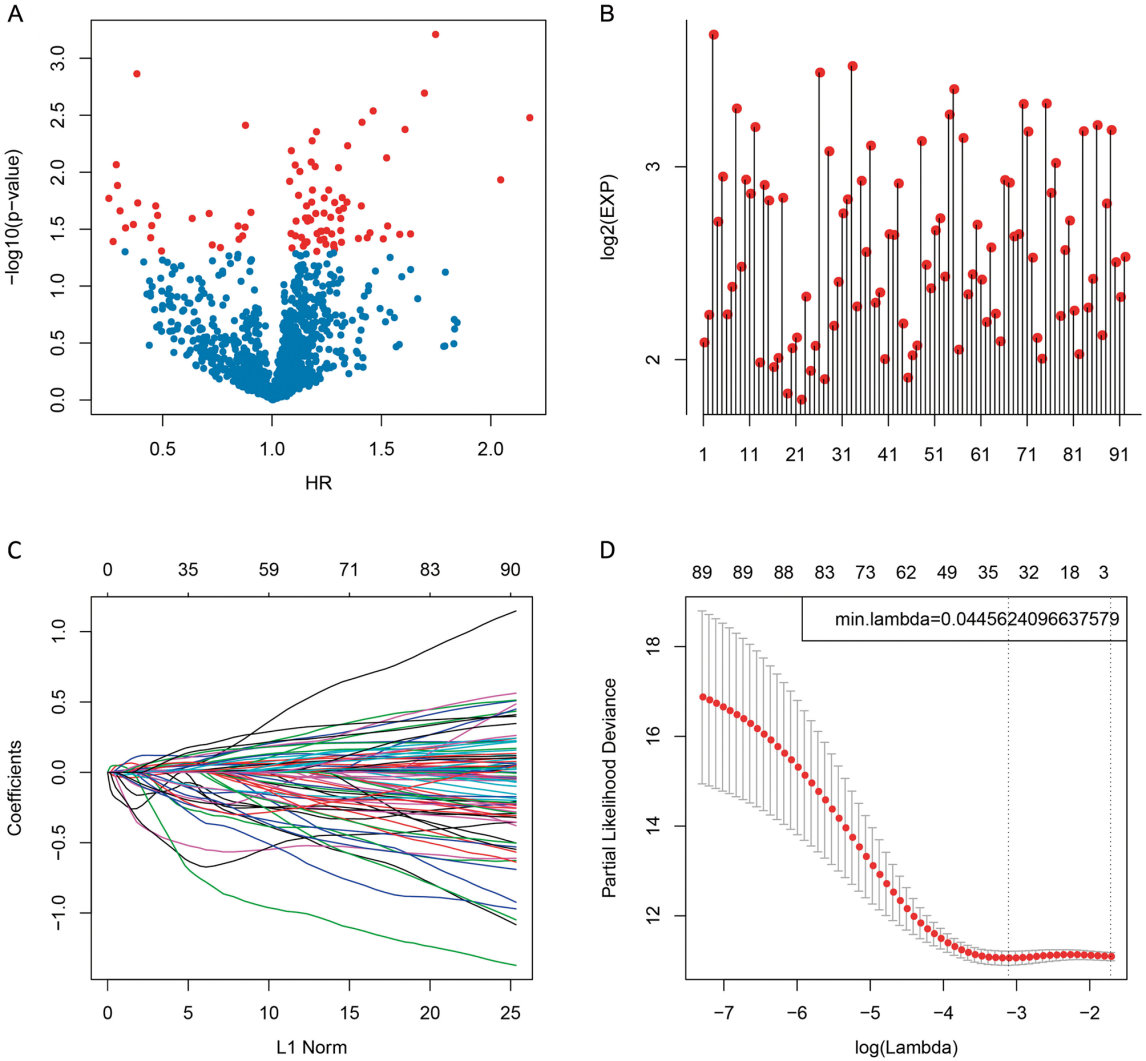
Construction of the prognosis prediction model for glioblastoma (GBM) patients by least absolute shrinkage and selection operator (LASSO) analysis. (**A**) The relationships between the *p *values of 92 genes and the hazard ratio (HR). (**B**) The relationships between the *p* values of 92 genes and the expression levels. Red dots represent significantly different immune-related genes regarding prognosis. (**C**) The changing trajectory of each independent variable. The horizontal axis represents the log value of the independent variable lambda, and the vertical axis represents the coefficient of the independent variable. With the increase in lambda, the number of independent variable coefficients tending to 0 also increases. (**D**) Confidence intervals for each lambda. The optimal model is acquired when the lambda is 0.04456.

At first, related data were collected based on the ImmPort and TCGA databases, followed by a pre-processing. Then, all immune-related genes and survival data were analyzed using the univariate Cox proportional hazards regression model based on the R survival package coxph function, with the significance level set at* p* < 0.05 (Supplementary Table S1). Finally, 92 prognosis-specific immune-related genes were mined. The association between the *p* values for these 92 genes and expression intensities (log2(EXP)), together with hazard ratios (HRs), is presented in Fig. 1A,B.

Altogether, 92 immune-related genes were identified, but most of them were not suitable for clinical detection. Therefore, the number of immune-related genes was reduced, while a high accuracy was maintained. Consequently, these 92 genes were narrowed down using a least absolute shrinkage and selection operator (LASSO) regression, to decrease the number of genes recruited into this risk model. The LASSO algorithm, a biased estimate used for processing multicollinearity data, can predict and select variables, and overcome the multicollinearity problem in regression analysis. Here, R package glmnet was utilized for LASSO regression analysis. The variation trajectory of each independent variable was assessed, as presented in Fig. 1C, which indicated that most independent parameters had coefficients of about zero with a gradual lambda increase. Moreover, the model was also established by means of a tenfold cross-validation. Figure 1D displays the confidence interval of each lambda, which reveals that the optimal model was acquired when the lambda was 0.04456. Therefore, this model was selected as the final model, involving 34 immune-related genes (Supplementary Table S2). Moreover, MASS R package was used for stepwise regression analysis, according to Akaike data standards, and 14 genes were used for the risk model construction (Supplementary Tables S3 and S4). The formula is presented in the “Methods” section.

### Construction of the model to predict prognosis for GBM patients

**Figure 2:**
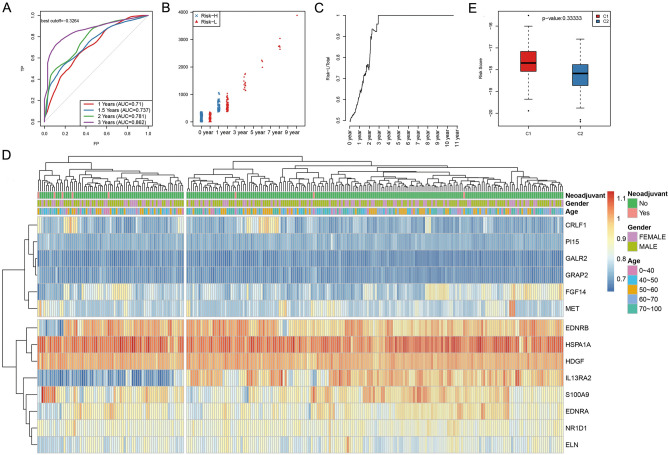
Verification of the stability of the prognosis prediction model including 14 immune-related genes of GBM patients in the training set. (**A**) The 1–3-year overall survival (OS) predicted receiver operating characteristic (ROC) curves of a 14-gene risk model in the training set. (**B**) The distribution of samples in Risk-H and Risk-L groups of the training set was done using the 14-gene risk model under different OS. (**C**) The level of Risk-L group/Total sample size with the extension in OS in the training set. (**D**) The clustering results of the training set samples. Fourteen genes were used for hierarchical clustering. The distance between different features was calculated by a Euclidean distance analysis. These genes clustered into high- and low-expression groups, and samples in the training set were also divided into two groups. (**E**) Difference in the RiskScore between the two groups, which had been clustered by the expression of 14 genes of training set samples.

Then, all samples in the training set were substituted into the formula to calculate the RiskScore value. The median RiskScore value was used as the threshold to classify patients into high- (Risk-H) and low-risk (Risk-L) groups. Receiver operating characteristic (ROC) analysis was also performed for prognosis classification according to the RiskScore value. The OS of all samples was 1–3 years (Supplementary Fig. S1). As a result (Fig. 2A), the model prediction efficiency for 1–3-year OS was examined, and the average area under the curve (AUC) was as high as 0.793. Moreover, Fig. 2B shows the sample distribution in Risk-H and Risk-L groups for various OS, suggesting no statistically significant differences in 0- and 1-year sample sizes between the two groups. Moreover, the 1.5-year sample size of Risk-H group was remarkably decreased compared with that of Risk-L group, which was more obvious with the OS extension (Fig. 2C). We next extracted the gene expression profile for the clustering analysis using log10 for all expression values. We also used the hierarchical clustering method to calculate the Euclidean distance between different features. Figure 2D shows the results of sample clustering of the training set. As expected, the above-mentioned 14 genes were markedly clustered into high and low expression groups, respectively, and the training set samples were also divided into two groups. Additionally, the RiskScore values between these two subclasses were compared (Fig. 2E).

To validate the model reliability, the expression patterns of the above 14 genes were extracted based on test cohort and substituted into the validation model. Meanwhile, the RiskScore values of all samples were also computed, and the test set data were also used to evaluate the model efficacy to predict the OS at 1–3 years, as presented in Supplementary Fig. S2, which displays the sample distribution in Risk-H and Risk-L groups at various OS. The difference in the distribution of 0–1-year sample size between the two groups was not statistically significant. Moreover, the 2-year sample size in the Risk-H group was also notably decreased compared with that in the Risk-L group, which was even obvious with the OS extension (Supplementary Fig. S2). Supplementary Figure S2 shows the results of sample clustering of the test cohort, as well as the different RiskScore values between these two subgroups.

Moreover, we retrieved the GSE74187 data set with prognosis follow-up information from the GEO database. The expression matrix of these 14 genes was extracted from the expression profile and the risk score of each sample was calculated using the same method. We evaluated the ROC risk score analysis, which indicated that the average AUC at 1, 2, and 3 years was 0.83 (Supplementary Fig. S3A). According to the median of the high-risk group, the prognosis was significantly worse than that of the low-risk group (Supplementary Fig. S3B), which was consistent with the training and test sets.

In addition, the expression patterns of 14 genes extracted based on all the above 523 samples were substituted into the model to calculate the RiskScore values to validate the model reliability and stability (Supplementary Fig. S4), which exhibits the results of sample clustering and different RiskScore values between these two subgroups. Overall, the RiskScore model established based on the expression patterns of 14 immune-related genes presented favorable accuracy and stability to identify immunity-related features.

**Figure 3:**
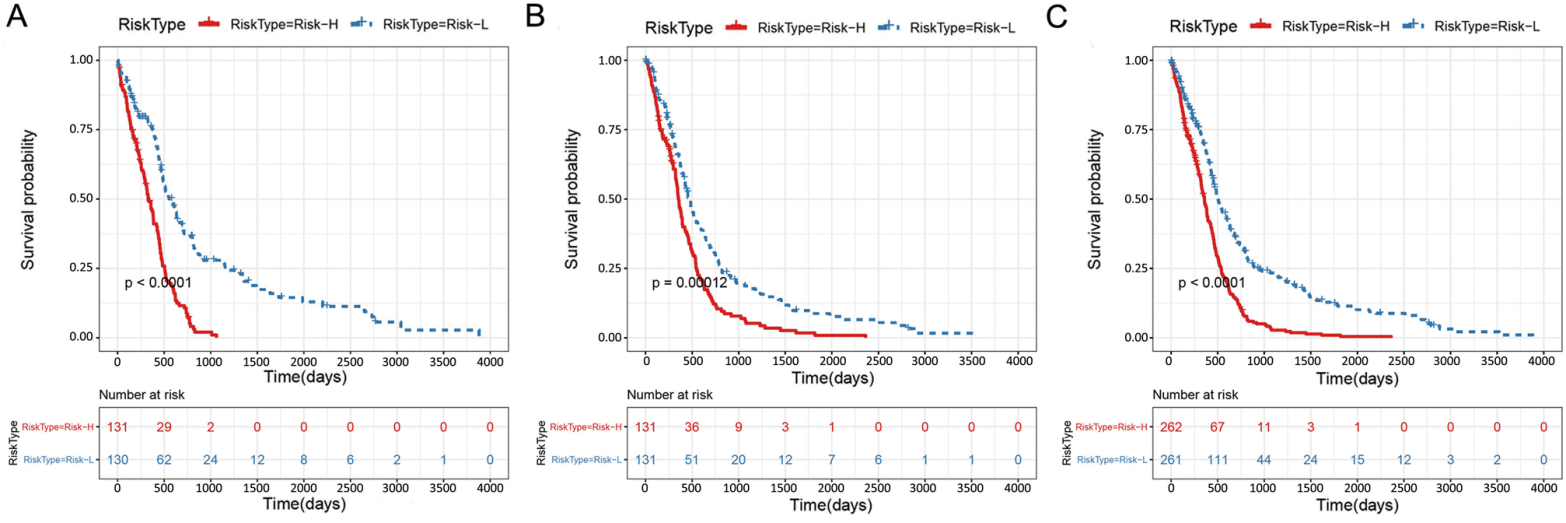
The Kaplan–Meier survival curve of the 14-gene-based risk model predicting the Risk-H and Risk-L groups in the training set (**A**, n = 261), test set (**B**, n = 262), and all samples (**C**, n = 523).

Finally, we plotted the Kaplan–Meier survival curves of the Risk-H and Risk-L groups based on the 14-gene-based risk model in the training (n = 261) and test cohorts (n = 262), and in all the samples (n = 523), separately, as shown in Fig. 3A–C (*p* < 0.0001, *p* < 0.001, and *p* < 0.0001, respectively).

### Functional annotations of immune-related genes and enrichment of signaling pathways specific to prognosis

All the above 14 gene families were first annotated according to the human gene classification in the HGNC database (Supplementary Table S5). All were significantly enriched in galanin receptors and endothelin receptor gene families (*p* < 0.05). Additionally, the clusterProfile in the R package was used for the enrichment analysis on the 14 genes. Supplementary Fig. S5 shows the results of the GO enrichment analysis and Supplementary Table S6 shows the related data, which indicated that most genes were enriched to distinct immune-related signaling pathways and biological processes.

**Figure 4:**
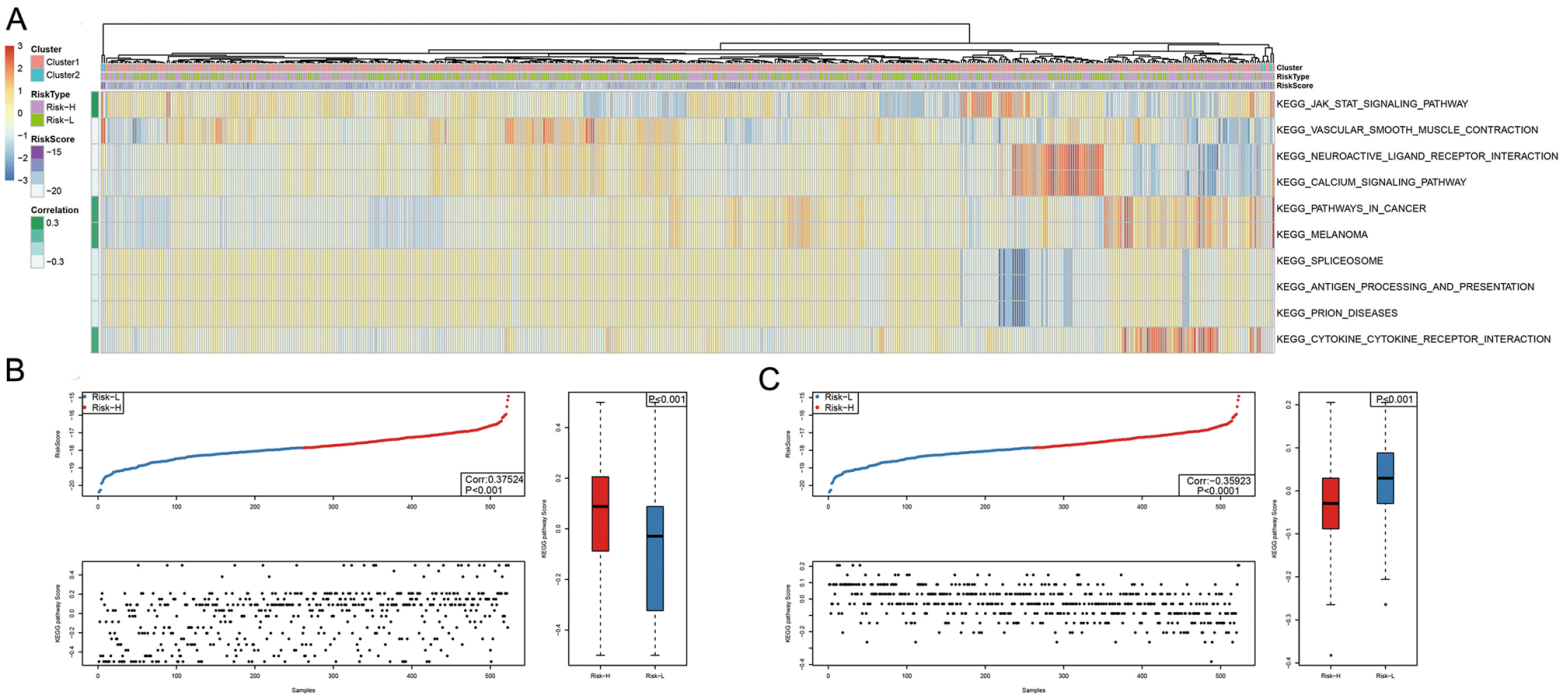
Correlation of RiskScore with signaling pathways. KEGG functional enrichment scores of each sample were analyzed and their correlation with RiskScore was calculated based on the enrichment score of each pathway in each sample. All 21 pathways related to the KEGG pathways are shown. (**A**) The clustering analysis was conducted according to the enrichment scores. (**B**) The distribution of JAK-STAT KEGG pathway enrichment scores in Risk-H and Risk-L groups for GBM patients. (**C**) Distribution of the vascular smooth muscle contraction KEGG pathway enrichment scores in Risk-H and Risk-L groups for GBM patients.

The R package GSVA ssGSEA function was used for KEGG functional enrichment. Associations with the RiskScore values were examined based on the pathway enrichment scores among the different samples to obtain a total of 21 KEGG-related pathways (Supplementary Table S7–S9). These 21 pathways were chosen for clustering analysis in accordance with the sample enrichment results from the training cohort (Fig. 4A). Additionally, the relationship between the enrichment score and the RiskScore value was examined by selecting the two major pathways with the highest GSEA enrichment scores (e.g., vascular smooth muscle contraction and the JAK-STAT signaling pathway). The sample distribution in the two groups was also explored. We found that the pathway enrichment scores were different in the Risk-H relative to the Risk-L group (Fig. 4B,C).

### Relationships of the RiskScore values with the clinical characteristics of samples

**Figure 5:**
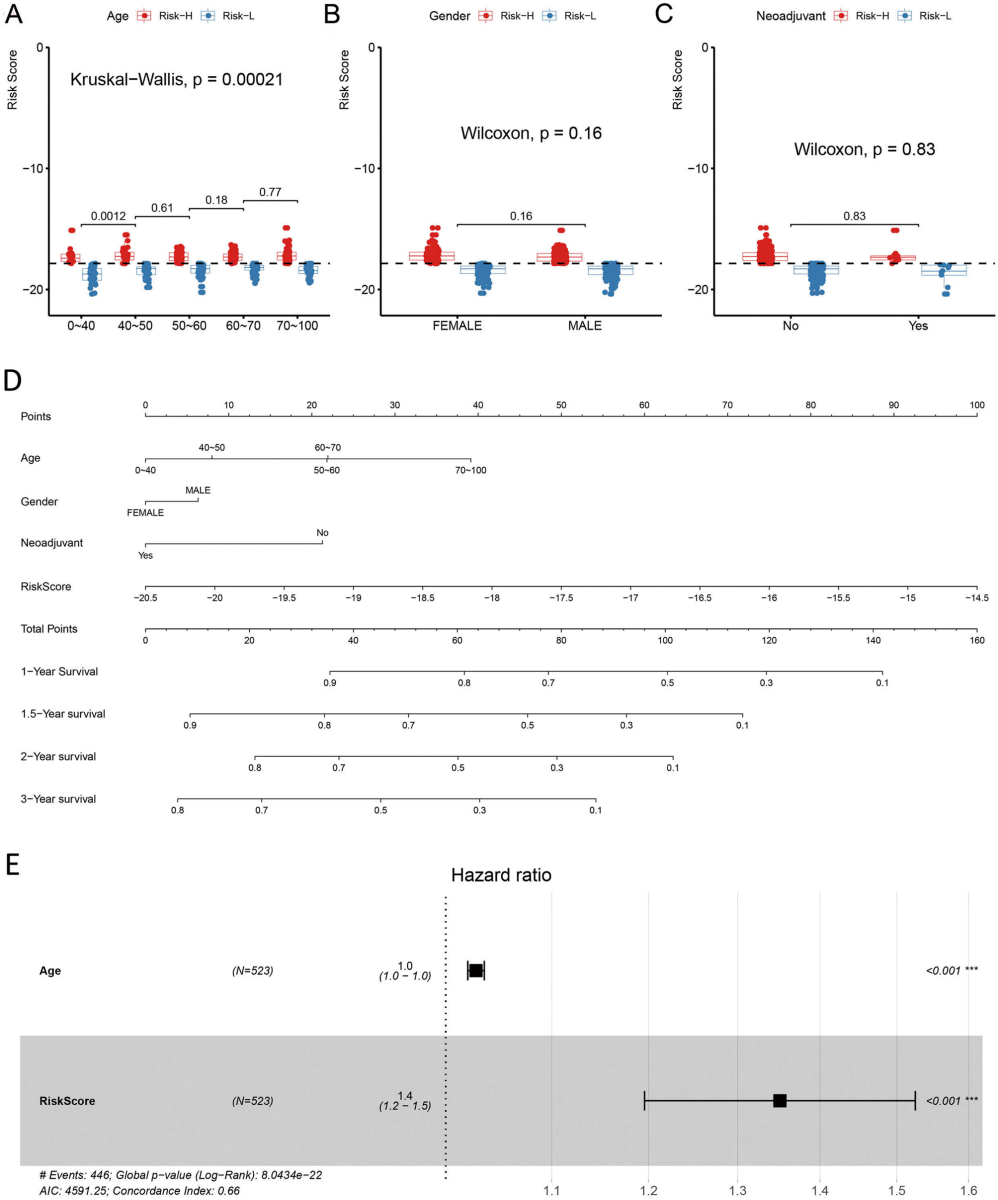
The relationships of different clinical factors with RiskScore values of GBM patients. Comparison of RiskScore among different ages (**A**), sexes (**B**), and neoadjuvants (**C**). The horizontal axis represents the different clinical factors, and the vertical axis represents RiskScore values. The constructed RiskScore model was dependent on patient age. (**D**) The nomogram model constructed by combining the clinical features (age, sex, neoadjuvant) with the RiskScore of GBM patients. There was an obvious association with the greatest influence on predicting the survival rate. (**E**) The forest plot constructed by combining age with RiskScore for GBM patients. The HR for RiskScore was approximately 1.4 in the forest plots established in combination with RiskScore and age (*p* < 0.05).

Subsequently, the associations between various parameters (such as neoadjuvant, sex, and age) and the RiskScore value were examined (Fig. 5A–C). Clearly, other features were not related to the RiskScore value (*p* > 0.05), except for age, and the constructed RiskScore model was dependent on patient age.

At last, the RiskScore values combined with the clinical characteristics were used to construct the nomogram model. Use of a nomogram, an approach to intuitively and effectively present risk model results, is convenient for predicting patient outcomes. Specifically, the straight-line length in a nomogram represents the effects of different parameters and their significance on the outcome. Here, a nomogram was constructed to combine the RiskScore, age, neoadjuvant, and sex, respectively, as displayed in Fig. 5D. RiskScore characteristics showed an obvious association with the greatest influence on predicting the survival rate, indicating that the 14-gene-based risk model had a superb prognosis prediction ability.

The forest plot was established based on the clinical characteristics and RiskScore. In Fig. 5E, the HRs for RiskScore were approximately 1.4 (*p* < 0.05).

Indeed, we also had analyzed the relationship between the expression level of 36 immune-checkpoint genes and RiskScore (Supplementary Table S10). In addition to eight genes, including *PDCD1* and *CTLA4*, the expression levels of other 26 genes showed a positive correlation with RiskScore, suggesting that the constructed model was able to instruct clinicians in diagnosing and predicting the prognosis for various immunophenotypes.

### Practical application of the prediction model for GBM patients

**Figure 6:**
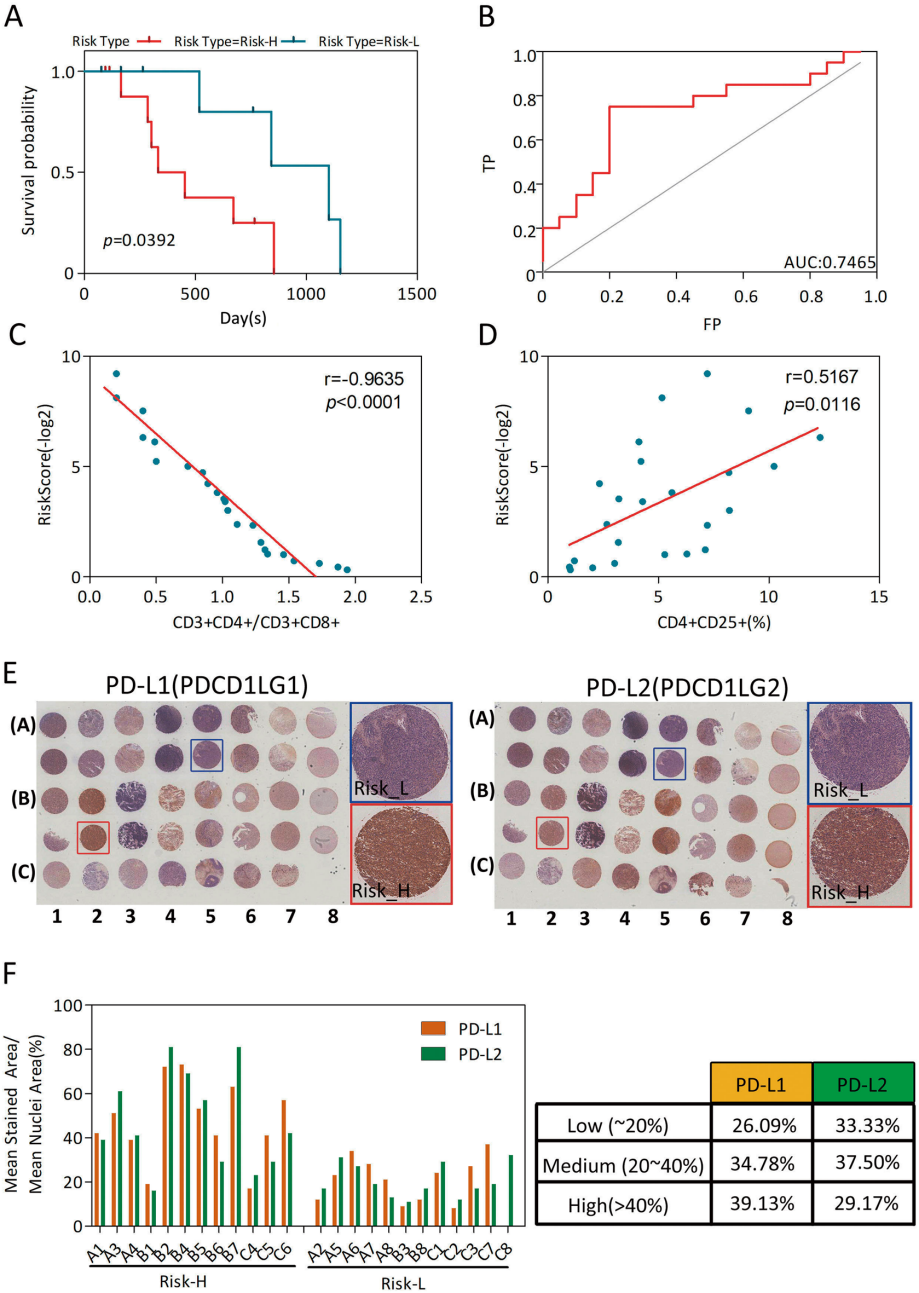
Clinical practice application of the prognostic predictor. (**A**) OS curves of the two clusters predicted from 24 GBM patients using the prognosis model. The log-rank test was used to assess the statistical significance of the difference. The red line indicates the Risk-H group, while the blue line indicates the Risk-L group, based on the median RiskScore value. (**B**) ROC curve with AUC under the final prognostic predictor. (**C**) Relationship between the RiskScore value and the score of CD3+CD4+/CD3+CD8+ cells of the peripheral blood samples of 24 GBM patients. The RiskScore value was negatively associated with the ratio of CD3+CD4+/CD3+CD8+ cells. (**D**) Relationship between the RiskScore value and the percentage of CD4+CD25+ Tregs in peripheral blood samples of the 24 GBM patients. The RiskScore value was positively related with the percentage of CD4+CD25+ Tregs. (**E**) Immunohistochemical (IHC) analysis of PD-L1 (left) and PD-L2 (right) for the 24 GBM patients. (**F**) Relationship between the IHC score of PD-L1 (yellow) or PD-L2 (green) and the RiskScore groups. The IHC score was positively correlated with the RiskScore value.

According to the prognostic prediction model, we analyzed the clinical follow-up data of these 24 GBM patients, which were divided into Risk-H and Risk-L groups (n = 12, each), based on the median RiskScore value. There was an inverse correlation between the RiskScore value and OS (*p* = 0.0392) (Fig. 6A), with an AUC of 0.7465 (Fig. 6B).

CD4+CD25+ regulatory T cells (Tregs) play an important role in anti-tumor immune responses, and a poor prognosis and declining survival rates are closely related with high Treg expression in cancer patients^14,15^. Consistent with these, the RiskScore value showed a negative relationship with CD3+CD4+/CD3+CD8+ (r = − 0.9635, *p* < 0.0001; Fig. 6C), but a positive relationship with CD4+CD25+ Tregs percentage (r = 0.5167, *p* = 0.0116; Fig. 6D). Notably, PD-L1 or PD-L2 immunohistochemical (IHC) analysis results showed that the IHC score was positively correlated with the RiskScore (Fig. 6E,F).

Taken together, we concluded that this prognostic predictor showed great promise in clinical practice application.

## Discussion

Currently, GBM treatments include surgery alone for an early-stage disease and adjuvant radio/chemotherapy plus surgical resection for an advanced stage. However, surgical resection cannot provide a satisfactory effect because cancer cells may have invaded the local adjacent tissues or developed metastasis^16^. Moreover, it is still controversial whether systemic adjuvant therapy can be prescribed following surgery owing to tumor heterogeneity or potential adverse effects^17^. Consequently, it is important to mine the potential biomarkers to predict GBM prognosis; this way, high-risk GBM cases can benefit from early adjuvant therapy. This can also assist in the clinical management of individual patients and thereby accurately distinguish patients that can be completely treated using adjuvant treatment from those that can avoid treatment and the possible chemotherapeutics-derived toxicity^18^. In the current work, a candidate signature was examined as a reliable method to predict GBM prognosis.

Due to the emerging next-generation sequencing techniques, a number of candidate biomarkers for the diagnosis and prognosis prediction of GBM were identified, which makes it possible to more specifically classify and more accurately predict GBM outcomes^19^. Several molecular markers, such as isocitrate dehydrogenase, O6-methylguanine DNA methyltransferase, phosphatase and tensin homolog, and epidermal growth factor receptor, are conventionally examined in clinical GBM cases^20,21^. These molecular markers facilitate targeted anti-GBM treatments and individualized therapeutic methods. Nonetheless, GBM has a dismal prognosis, so new treatment strategies and molecular biomarkers are urgently needed to illustrate the underlying GBM mechanisms and improve the OS of patients.

Limited clinical data and fresh tumor specimens symbolizing transitional steps from tumor initiation to progression are important barriers to improving clinical outcomes in GBM patients. Methylation-based subtypes that predict GBM patient survival have been reported. Notably, the methylation levels of different subgroups could reflect different molecular genetic features^22,23^. More and more attention has been paid to the relationship between the immune system and malignancy progression and pathogenesis, which contribute to GBM treatment, thereby promoting the development of anti-tumor treatments. CD68+ and CD163+ cells were the most abundant populations in GBM, and the percentage of CD163+ cells correlated with a poorer prognosis. Mesenchymal GBMs displayed the highest percentages of microglia, macrophage, and lymphocyte infiltration^24^. Wild-type and the mesenchymal subtype, IDH1, in GBM presented strong immunosuppressive microenvironments, while tumors of mutated IDH1 and TCGA proneural subtypes exhibited a significantly less immunosuppressive state^25^. Regarding tumor origin (namely, the immune system), the approach of regulating and killing cancer cells by modulating the immune system and promoting anti-cancer immunity in the tumor microenvironment is novel. Therefore, screening of novel significant prognosis-specific immune-related genes is meaningful for predicting disease prognosis and identifying novel therapeutic targets. Some researchers have reported gene expression-based immunoprofiling of GBM using TCGA data. For example, Arivazhagan et al. reported a 14-gene expression signature that predicted survival in GBM patients. A network analysis specifically revealed inflammatory response pathway activation in the high-risk group^26^. Zhang et al. showed that samples with high tumor microenvironment (TME) scores were characterized by immune activation, TGF pathway activation, and high expression of immune checkpoint genes, while those with low TME scores were characterized by a high-frequency of *IDH1* and *MET* mutations^27^. Zhang et al. identified six immune-related genes (*CANX, HSPA1B, KLRC2, PSMC6, RFXAP,* and *TAP1*) as risk signatures. Importantly, Kaplan–Meier and ROC curves, as well as risk plotting, verified their performance in TCGA and CGGA datasets^28^. Zhang et al. observed that a high immune score was associated with low methylation and copy number variation levels, a high expression of immunosuppressive markers (CD27, PDL1 and CTLA4), and a shorter recurrence-free survival^29^. Here, GBM classification based on the prognosis-specific and immune-related signature could precisely estimate the clinical outcomes and identify those with a high or low risk of postoperative recurrence. Notably, PD-L1 or PD-L2 IHC analysis results showed that the IHC score was positively correlated with the RiskScore. Moreover, the RiskScore value showed a negative relationship with CD3+CD4+/CD3+CD8+, but a positive relationship with CD4+CD25+ Tregs percentage.

Here, 14 prognosis-specific immune-related genes were mined by big data mining, TCGA and ImmPort database sorting, and statistical analyses. Two key points must be cautiously taken into consideration to ensure the prognosis model validity: clinical utility and transport capability in different cohorts. Typically, our constructed prognosis model is better than other prognosis models for GBM that were not duplicated in GBM-independent cohorts. Additionally, our validation set was a multi-institutional cohort involving cases from different hospitals, which suggests that our constructed GBM model is applicable to different clinical settings and patient types. Afterwards, the 14-gene-based model was constructed for prognosis prediction, and RiskScore values for all cases were also computed. Then, the model was applied for prediction and validation. The prognosis model was established based on the expression patterns of specific immune-related genes, and it could classify patients at a certain clinical stage into various subgroups, according to the estimated survival outcomes.

Nine of these 14 genes were previously suggested to be involved in malignant transformation, pathogenesis, progression, and immune microenvironment of GBM, including *S100A9*, *HSPA1A*, *GALR2*, *EDNRB*, *IL13RA2*, *ELN*, *NR1D1*, *HDGF*, and *MET*^30–35^. They were markedly correlated with patient survival and prognosis, which means that our bioinformatic mining displayed a high reliability and accuracy. However, the relationship of the other two genes (namely, *CLRF1* and *GRAP2*) with GBM is not validated in a clinical or basic study, and we are interested in this topic. *CRLF1* is verified to be involved in regulating malignant cancer cell proliferation and invasion, which can affect signaling pathways (such as MAPK/ERK and Akt/PI3K) and modulate the immune and nervous systems maturity during fetal development^36,37^. *GRAP2* is also found to be a candidate tumor suppressor, and it is recognized to be a prognosis prediction marker for different types of cancers, which can regulate tumor cell sensitivity to immunotherapy^38,39^.

In conclusion, our results assist in identifying novel biomarkers for predicting the clinical prognosis of GBM. Additionally, the 14-gene-based risk model can provide a variety of targets for an accurate GBM treatment, and it can also help classify GBM patients according to the molecular subtypes. In addition, the constructed model may be used to instruct clinicians in the medication, prognosis prediction, and diagnosis of GBM patients with various immunophenotypes.

## Methods

GBM tissue specimens were collected from 24 patients (ages 42–75) who underwent curative resection for glioma with informed consent between 2017 and 2019 at Hefei Cancer Hospital, Chinese Academy of Sciences (CAS), with Institutional Review Board approval. All methods were performed in accordance with the relevant guidelines and regulations, as stated in relevant sections below.

### Pre-processing of original sample data and preliminary selection of immune-related genes in GBM

The up-to-date clinical follow-up information was extracted from TCGA GDC API. Altogether, 539 RNA-Seq data samples were mined (as displayed in Supplementary Table S11), and 529 of them were tumor tissues. Additionally, the immune-related gene set involving 1811 genes was also acquired based on the ImmPort database^40^ (Supplementary Table S12).

At first, 529 tumor tissues were subjected to a pro-processing (Supplementary Table S13), and 523 of them involving 1,108 genes were used for further model analysis. Supplementary Table S14 presents the clinical characteristics of samples. Afterwards, these 523 samples were classified into training and test sets, respectively. Random grouping with replacement was carried out 100 times on all samples to remove the influence of random allocation bias on model stability. The training (n = 261) and test set (n = 262) samples are displayed in Supplementary Tables S15 and 16, respectively. The eventual data of training and test set samples are shown in Supplementary Table S14. Differences between the two sets were not statistically significant, indicating a reasonable sample grouping.

### Univariate survival analysis for immune-related samples in training set

The univariate Cox proportional hazards regression model was utilized to analyze the immune-related genes and the survival data using the survival coxph function^41^ of R package. A *p* < 0.05 was regarded to be statistically significant.

### Screening of immune-related genes specific to GBM prognosis, and establishment of the model to predict prognosis

At first, the R package MASS and glmnet functions were used for stepwise and LASSO regression analysis^42^, and the risk model was established based on specific immune-related genes, as displayed below:

1 $$ RiskScore = EDNRA \times -0.325652748 + HSPA1A \times -0.312268258 + S100A9 \times 0.17460672 + PI15 \times -1.128026913 + EDNRB \times -0.199258031 + GALR2 \times -1.690737959 + NR1D1 \times 0.367374589 + FGF14 \times 0.184640626 + ELN \times 0.258161826 + IL13RA2 \times 0.081069744 + MET \times 0.172446326 + HDGF \times -0.342300085 + GRAP2 \times -0.863180168 + CRLF1 \times -0.138403709 $$

Afterwards, related gene expression patterns were selected based on training and test sets, which were then substituted into the constructed model to calculate the RiskScore values in each sample. The median RiskScore value was utilized as the threshold to classify samples as belonging to the high- (Risk-H) or low-risk (Risk-L) group. Finally, the accuracy, stability, and efficiency of the model to predict and classify GBM prognosis were evaluated through gene clustering, ROC, and KM analyses.

### Signaling pathway enrichment and functional annotations for immune-related genes specific to immunity

Finally, 14 genes were screened and the corresponding gene families were annotated in accordance with the human gene classification in the HGNC database^43^. Moreover, GO enrichment analyses were carried out using these 14 prognosis-specific immune-related genes and clusterProfile^44^ of R package.

### Relationships of RiskScore with the signaling pathways and clinical characteristics of samples

At first, the R package GSVA^45^ ssGSEA function was utilized to evaluate the score of KEGG enrichment analysis. At the same time, the relationship of RiskScore was computed, and later, clustering analysis was performed based on the pathway enrichment score for all samples. Then, the relationships of related factors (like neoadjuvant, sex, and age) with the RiskScore were determined. Finally, the nomogram model was established, and related clinical characteristics and RiskScore values were used to draw the forest plot, and the relationships between RiskScore and clinical characteristics with patient survival were examined.

### Phenotyping of peripheral T cells and IHC staining for GBM tissue microarray analysis

Peripheral blood samples from 24 GBM patients undergoing curative resection with informed consent between 2017 and 2019 at Hefei Cancer Hospital, Chinese Academy of Sciences (Anhui, China), were stained with the following sets of monoclonal antibodies (BD Biosciences; San Jose, CA, USA): CD3-PE (clone SP34), CD4-APC-Cy7 (clone SK3), CD8-PerCP (clone SK1), and CD25-FITC (clone MA251), and analyzed on Cytomics FC500 Flow Cytometer CXP with the CXP analysis software (Beckman Coulter Inc.). Twenty-four GBM tissues were placed on a tissue microarray and stained with anti-PD-L1 (clone E1L3N) and anti-PD-L2 (clone D7U8C) antibodies (Cell Signaling Technology; Danvers, MA, USA) , and visualized using the KF-PRO Digital Slide Scanning System (Kongfong Biotech International Co., LTD; Ningbo, China).

### Statistical methods

The TCGA dataset was randomly divided into training and test cohorts in a 1:1 ratio. Samples in the training set were analyzed to identify the potential prognosis-predicting genes and validated in both the test and the whole sets. First, the relationships between the expression of immune-related genes and patient OS were evaluated using the univariate Cox proportional hazards regression analysis. Typically, genes with a *p* < 0.05 through log rank test were selected to be the candidate variables. Later, the number of candidate genes was decreased based on the LASSO-Cox method, and later, immune-related genes showing the greatest significance were chosen for constructing the RiskScore model to predict prognosis. The RiskScore model could be calculated as follows:

2 $$Riskscore= \sum_{i=0}^{n}{\upbeta }_{i} \times {\upchi }_{i} $$

where β_i_ indicates the coefficient, and χ_i_ represents the gene expression level (fpkm) of each gene. The RiskScore model was calculated for all patients, who were then divided into low- or high-risk groups according to the median RiskScore value in the training set. Patients in the low-risk group had a lower risk of OS, while those in the high-risk group had a higher risk of OS. Then, the difference in OS between these two groups was calculated based on the Kaplan–Meier survival curve. The specificity and sensitivity of the model in diagnosis and prognosis prediction were evaluated according to the areas under the ROC curve. A two-tailed *p* < 0.05 was deemed to indicate statistical significance. The Bio-conductor and R software (version 3.5.0) were utilized for all statistical analyses.

### Ethics approval and consent to participate

This study was reviewed and approved by the Institutional Review Board of the Cancer Hospital of Hefei Institutes of Physical Science, CAS, and written informed consent was obtained from patients based on the Declaration of Helsinki.

## Supplementary information

Supplementary figures.

Supplementary tables.

Supplementary legends.

## Abbreviations

AUC: Area under the curve
GO: Gene ontology
GBM: Glioblastoma
HRs: Hazard ratios
HGNC: HUGO Gene Nomenclature Committee
IDH1: Isocitrate dehydrogenase (NADP(+)) 1
KEGG: Kyoto Encyclopedia of Genes and Genomes
LASSO: Least absolute shrinkage and selection operator
MET: MET proto-oncogene, receptor tyrosine kinase
OS: Overall survival
ROC: Receiver operating characteristic
TGF: Transforming growth factor
