# Supplementary material for: Molecular subtyping of glioblastoma based on immune-related genes for prognosis
Source: Sci Rep. 2020 Sep 23;10:15495. doi: 10.1038/s41598-020-72488-4 (PMC7511296; doi:10.1038/s41598-020-72488-4)
Supplement: Supplementary file 1 — Supplementary figures. [file 41598_2020_72488_MOESM1_ESM.pdf]

Supplementary Fig. S1

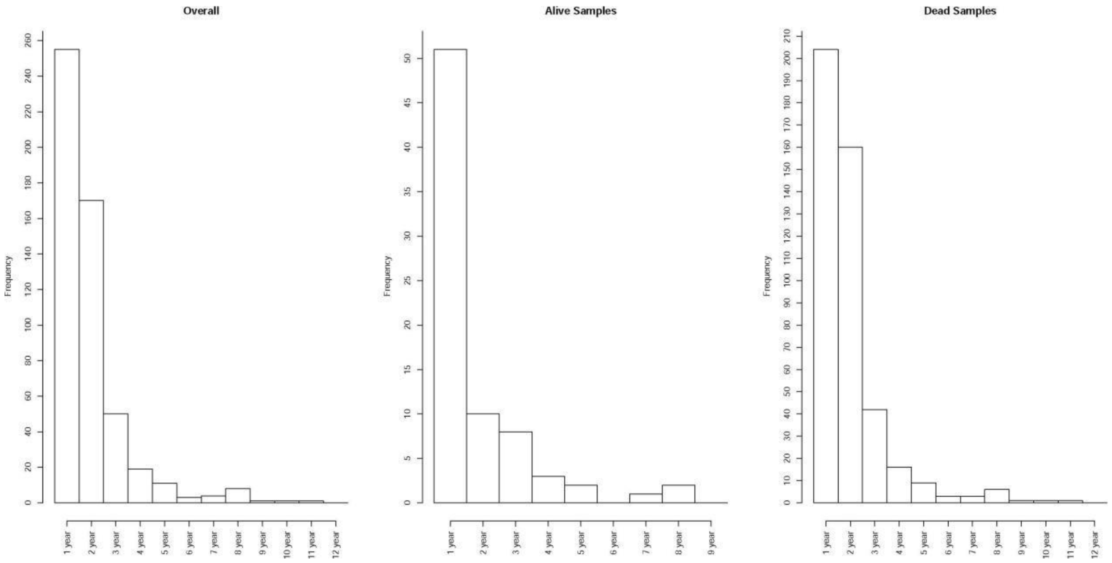

Supplementary Fig. S1. The distribution of follow-up period in GBM samples (overall samples, alive samples and dead samples).

Supplementary Fig. S2

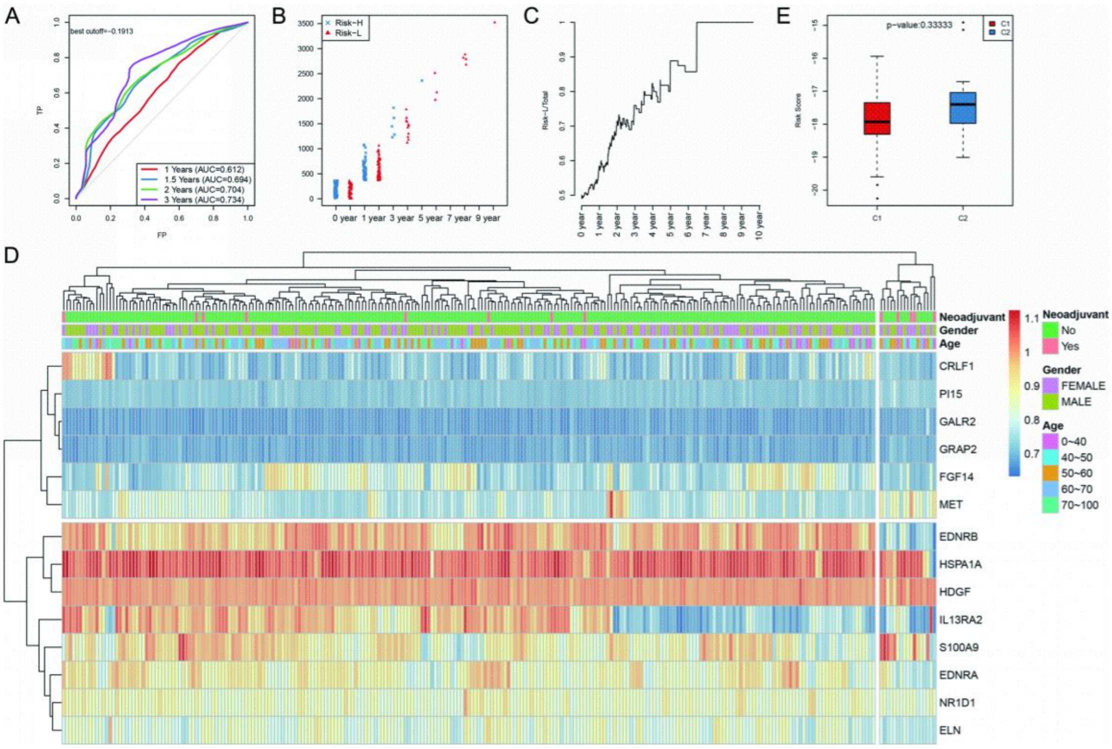

Supplementary Fig. S2. Verify the reliability of the prognosis prediction model included 14 immune-related genes for GBM patients in test set. (A) The survival predicted ROC curves of 14-gene risk model in test set. (B) The distribution of samples in Risk-H and Risk-L groups of test set divided through 14-gene risk model under different OS. (C) The level of Risk-L group/Total sample size with the extension in OS in the test set. (D) The clustering results of test set samples. (E) Difference in the RiskScore between the two groups which had been clustered by the expression of 14 genes of test set samples.

Supplementary Fig. S3

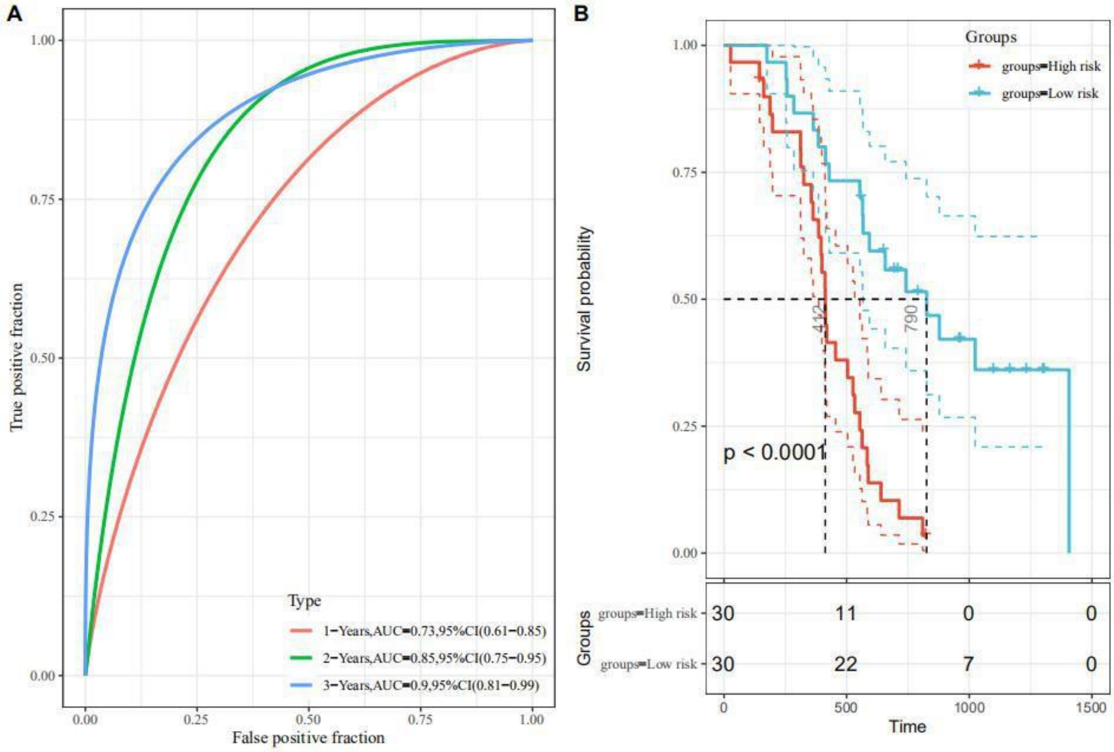

Supplementary Fig. S3. Verification of the reliability of the prognosis prediction model including 14 immune-related genes for GBM patients in the GEO database GSE74187**.** (A) The survival prediction ROC curves of the 14-gene risk model in the GSE74187 set. (B) The distribution of samples in Risk-H and Risk-L groups of the GSE74187 set was done using the 14-gene risk model under different OS.

Supplementary Fig. S4

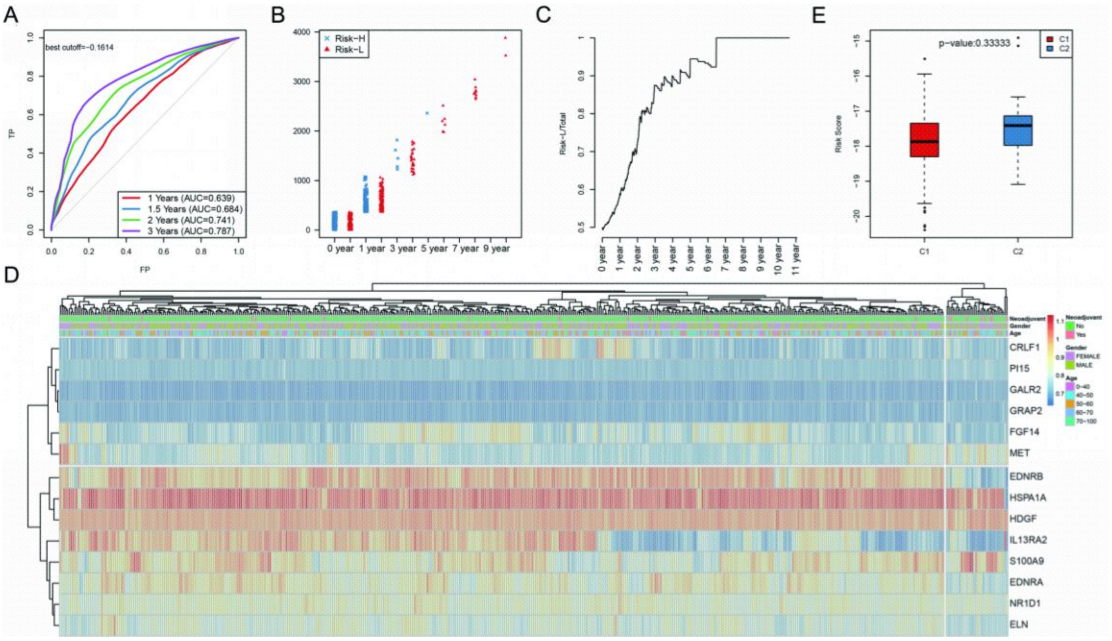

Supplementary Fig. S4. Verify the stability of the prognosis prediction model included 14 immune-related genes for all the samples. (A) The survival predicted ROC curves of 14-gene risk model. (B) The distribution of samples in Risk-H and Risk-L groups divided through 14-gene risk model under different OS. (C) The level of Risk-L group/Total sample size with the extension in OS. (D) The clustering results of all the samples. (E) Difference in the RiskScore between the two groups which had been clustered by the expression of 14 genes.

Supplementary Fig. S5

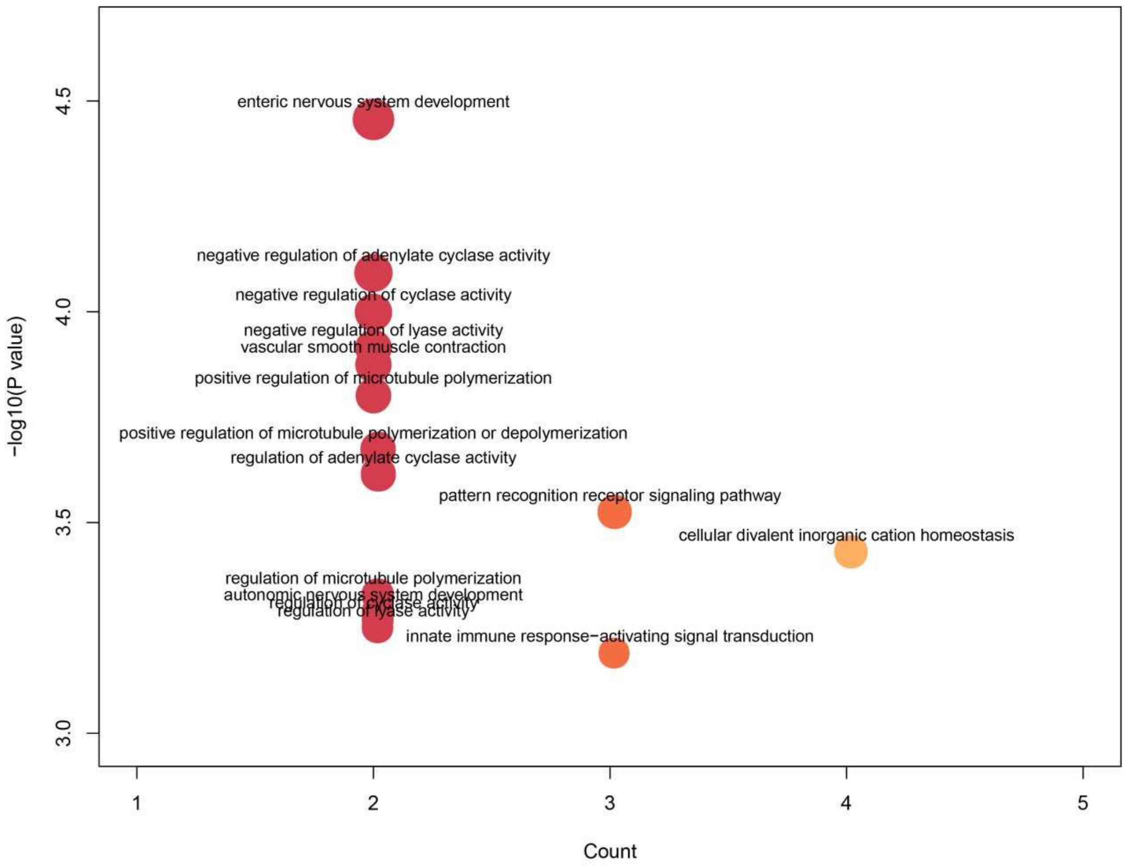

Supplementary Fig. S5. The GO enrichment analysis of the 14 specific immune-related genes.
