# Supplementary material for: Molecular subtyping of glioblastoma based on immune-related genes for prognosis
Source: Sci Rep. 2020 Sep 23;10:15495. doi: 10.1038/s41598-020-72488-4 (PMC7511296; doi:10.1038/s41598-020-72488-4)
Supplement: Supplementary file 3 — Supplementary legends. [file 41598_2020_72488_MOESM3_ESM.docx]

**Supplementary data**

**Molecular Subtyping of Glioblastoma Based on Immune-related Genes for Prognosis**

Xueran Chen ^1,2,#,*^, Xiaoqing Fan ^3,4,#^, Chenggang Zhao ^1,5^, Zhiyang Zhao ^1,5^, Lizhu Hu ^1,5^, Delong Wang ^3,4^, Ruiting Wang ^3,4^, and Zhiyou Fang ^1,2^

^1^ Anhui Province Key Laboratory of Medical Physics and Technology; Institute of Health and Medical Technology, Hefei Institutes of Physical Science, Chinese Academy of Sciences, No. 350, Shushan Hu Road, Hefei, Anhui, 230031, China

^2^ Department of Molecular Pathology, Hefei Cancer Hospital, Chinese Academy of Sciences, No. 350, Shushan Hu Road, Hefei, Anhui, 230031, China

^3^ The First Affiliated Hospital of USTC, Division of Life Sciences and Medicine, University of Science and Technology of China (USTC), No. 17, Lujiang Road, Hefei, Anhui, 230001, China

^4^ Department of Anesthesiology, Anhui Provincial Hospital, No. 17, Lujiang Road, Hefei, Anhui, 230001, China

^5^ University of Science and Technology of China, No. 96, Jin Zhai Road, Hefei, Anhui, 230026, China

^#^ Co-first author

^*^Correspondence: [xueranchen@cmpt.ac.cn](mailto:xueranchen@cmpt.ac.cn) (X.R.C.)

**Supplementary Fig. S1. The distribution of follow-up period in GBM samples (overall, alive, and dead samples).**

**Supplementary Fig. S2. Verification of the reliability of the prognosis prediction model including 14 immune-related genes for GBM patients in the test set.** (A) The survival prediction ROC curves of the 14-gene risk model in the test set. (B) The distribution of samples in Risk-H and Risk-L groups of the test set was done using the 14-gene risk model under different OS. (C) The level of Risk-L group/Total sample size with the extension in OS in the test set. (D) The clustering results of test set samples. (E) Difference in the RiskScore between the two groups, which had been clustered by the expression of 14 genes in the test set samples.

**Supplementary Fig. S3. Verification of the reliability of the prognosis prediction model including 14 immune-related genes for GBM patients in the GEO database** GSE74187**.** (A) The survival prediction ROC curves of the 14-gene risk model in the GSE74187 set. (B) The distribution of samples in Risk-H and Risk-L groups of the GSE74187 set was done using the 14-gene risk model under different OS.

**Supplementary Fig. S4. Verification of the stability of the prognosis prediction model including 14 immune-related genes for all the samples.** (A) The survival prediction ROC curves of the 14-gene risk model. (B) The distribution of samples in Risk-H and Risk-L groups divided using the 14-gene risk model under different OS. (C) The level of Risk-L group/Total sample size with the extension in OS. (D) The clustering results of all the samples. (E) Difference in the RiskScore between the two groups, which had been clustered by the expression of 14 genes.

**Supplementary Fig. S5. The GO enrichment analysis of the 14 specific immune-related genes.**

**Supplementary Table S1. The relationship between the expression level of immune-related genes and prognosis based on the univariate Cox proportional hazards regression model.**

**Supplementary Table S2. The 34 immune-related genes in the risk model when the model’s lambda was 0.04456.**

**Supplementary Table S3. The 14 immune-related genes in the risk model after stepwise regression analysis with the R package MASS.**

**Supplementary Table S4. The 14 immune-related genes in the risk model after logical analysis.**

**Supplementary Table S5. The 14 gene families annotated according to the human gene classification in the HGNC database.**

**Supplementary Table S6. GO enrichment analysis of the 14 immune-related genes.**

**Supplementary Table S7. KEGG pathway analysis of the 14 immune-related genes in the training set.**

**Supplementary Table S8. KEGG pathway analysis of the 14 immune-related genes in the test set.**

**Supplementary Table S9. KEGG pathway analysis of the 14 immune-related genes with all GBM samples.**

**Supplementary Table S10. Relationship between immune-checkpoint genes and RiskScore.**

**Supplementary Table S11. The clinical follow-up information of 539 samples extracted from TCGA GDC API.**

**Supplementary Table S12. The immune-related gene set extracted from the ImmPort database.**

**Supplementary Table S13. The clinical follow-up information subjected to pro-processing.**

**Supplementary Table S14. The clinical characteristics of samples subjected to pro-processing.**

**Supplementary Table S15. The clinical follow-up information of the training set.**

**Supplementary Table S16. The clinical follow-up information of the test set.**
